# Supplementary material for: Netcombin: An algorithm for constructing optimal phylogenetic network from rooted triplets
Source: PLoS One. 2020 Sep 18;15(9):e0227842. doi: 10.1371/journal.pone.0227842 (PMC7500971; doi:10.1371/journal.pone.0227842)
Supplement: S4 File — (ZIP) [file pone.0227842.s004.zip › SequenceData-Triplet/sequenceData-10-20-40-Triplet2/data-(sample 10- length 500- num seq 40- m rate 10^-6)/5 seq.pdf]

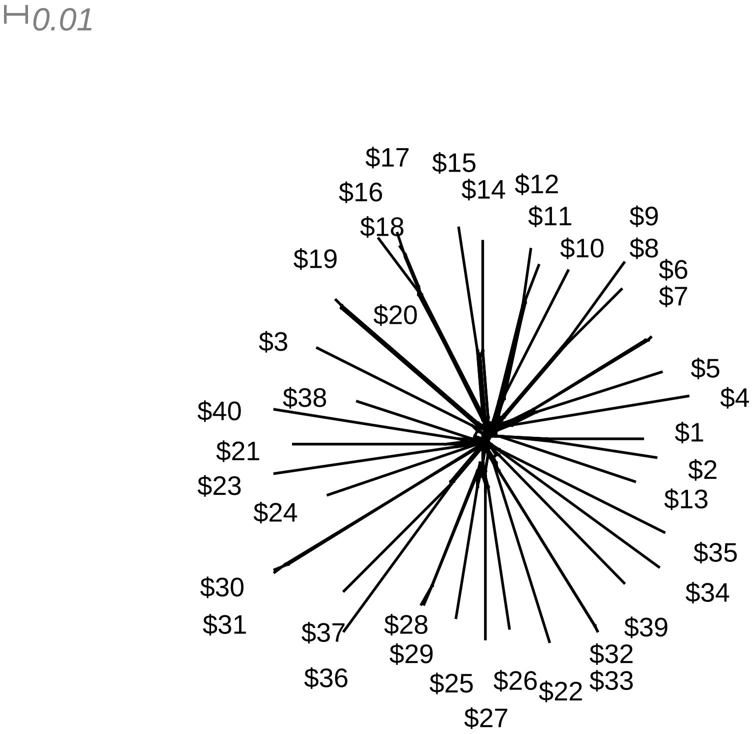
0.01
$17
$15
$16
$14
$12
$18
$11
$9
$10
$8
$19
$6
$7
$20
$3
$5
$38
$4
$40
$1
$21
$2
$23
$13
$24
$35
$30
$34
$31
$37
$28
$39
$29
$32
$36
$25
$26
$22
$33
$27
